# Genetic variants of *HIF1α* are associated with right ventricular fibrotic load in repaired tetralogy of Fallot patients: a cardiovascular magnetic resonance study

**DOI:** 10.1186/s12968-019-0555-2

**Published:** 2019-08-19

**Authors:** Thanh T. Hoang, Paulo Henrique Manso, Sharon Edman, Laura Mercer-Rosa, Laura E. Mitchell, Anshuman Sewda, Michael D. Swartz, Mark A. Fogel, A. J. Agopian, Elizabeth Goldmuntz

**Affiliations:** 1 Department of Epidemiology, Human Genetics, and Environmental Sciences, UTHealth School of Public Health, Houston, TX USA; 2 Department of Pediatrics, Ribeiro Preto Medical School USP, Ribeirao Preto, Brazil; 3 Division of Cardiology, Children’s Hospital of Philadelphia, Abramson Research Center 702A, 3615 Civic Center Boulevard, Philadelphia, PA 19104 USA; 4 Department of Pediatrics, University of Pennsylvania Perelman School of Medicine, Philadelphia, PA USA; 5 Department of Genetics and Genomic Sciences, Icahn School of Medicine at Mount Sinai, New York, NY USA; 6 Department of Biostatistics and Data Science, UTHealth School of Public Health, Houston, TX USA

**Keywords:** Cardiovascular magnetic resonance imaging, *HIF1α*, Tetralogy of Fallot, Fibrosis, Right ventricular ejection fraction, Right ventricular end-diastolic volume

## Abstract

**Background:**

Studies suggest that right ventricular (RV) fibrosis is associated with RV remodeling and long-term outcomes in patients with tetralogy of Fallot (TOF). Pre-operative hypoxia may increase expression of hypoxia inducible factor-1-alpha (*HIF1α*) and promote transforming growth factor β1 (*TGFβ1*)-mediated fibrosis. We hypothesized that there would be associations between: (1) RV fibrosis and RV function, (2) *HIF1α* variants and RV fibrosis, and (3) *HIF1α* variants and RV function among post-surgical TOF cases.

**Methods:**

We retrospectively measured post-surgical fibrotic load (indexed volume and fibrotic score) from 237 TOF cases who had existing cardiovascular magnetic resonance imaging using late gadolinium enhancement (LGE), and indicators of RV remodeling (i.e., ejection fraction [RVEF] and end-diastolic volume indexed [RVEDVI]). Genetic data were available in 125 cases. Analyses were conducted using multivariable linear mixed-effects regression with a random intercept and multivariable generalized Poisson regression with a random intercept.

**Results:**

Indexed fibrotic volume and fibrotic score significantly decreased RVEF by 1.6% (*p* = 0.04) and 0.9% (*p* = 0.03), respectively. Indexed fibrotic volume and score were not associated with RVEDVI. After adjusting for multiple comparisons, 6 of the 48 *HIF1α* polymorphisms (representing two unique signals) were associated with fibrotic score. None of the *HIF1α* polymorphisms were associated with indexed fibrotic volume, RVEDVI, or RVEF.

**Conclusion:**

The association of some *HIF1α* polymorphisms and fibrotic score suggests that *HIF1α* may modulate the fibrotic response in TOF.

**Electronic supplementary material:**

The online version of this article (10.1186/s12968-019-0555-2) contains supplementary material, which is available to authorized users.

## Introduction

Tetralogy of Fallot (TOF) is one of the most common types of severe congenital heart defects [1]. The majority of infants with repaired TOF reach adulthood [2, 3]. Indeed, there are now more adults than children living with TOF. However, patients with TOF often experience significant morbidity and early mortality due to residual pulmonary insufficiency, right ventricular (RV) dilation, and RV dysfunction [4].

Despite similar cardiac anatomy and surgical intervention, patients with TOF experience disparate outcomes that may be partially explained by individual responses to physiologic stresses (e.g., pre-operative hypoxia). Post-operative scarring (i.e., fibrosis) is a well-recognized consequence of TOF repair, particularly at the site of a ventriculotomy [5, 6]. Recent studies have identified additional areas of myocardial fibrosis using cardiovascular magnetic resonance imaging (CMR) and have demonstrated that fibrotic load varies in amount. Early studies have suggested that myocardial fibrosis is associated with RV volume load and longer cardiopulmonary bypass times, and that myocardial fibrosis may contribute to RV dysfunction [5, 7–12]. However, the relationship of RV fibrosis to RV remodeling and clinical outcome is incompletely understood.

One hypothetical cause of RV fibrosis in TOF is early exposure to hypoxia and consequential upregulation of hypoxia response genes. Prior to surgery, neonates or infants with TOF are hypoxic for days to months, which may trigger increased expression of hypoxia-inducible factor 1α (*HIF1α*). Studies have shown that chronic up-regulation of *HIF1α* promotes transforming growth factor β1 (*TGFβ1*)-mediated fibrosis [13, 14]. *TGFβ1* transforms endothelial and smooth muscle cells into fibroblasts [15]. Under stress, fibroblasts convert to myofibroblasts. Myofibroblasts induce extracellular matrix deposition, resulting in fibrosis. Jeewa et al. hypothesized that variants of *HIF1α* modulate the extent of fibrosis in patients with TOF [16] and reported that three *HIF1α* variants (rs10873142, rs2057482 and rs11549465) were associated with RV fibrosis at the time of surgical repair, as well as RV function and RV dilation at follow-up [16]. These findings suggest that variants of *HIF1α* may modulate the extent of RV fibrosis and subsequent function in patients with TOF. However, their study did not report a comprehensive assessment of the *HIF1α* gene or consider the extent of RV fibrosis as measured by CMR following surgical repair.

**Fig. 1:**
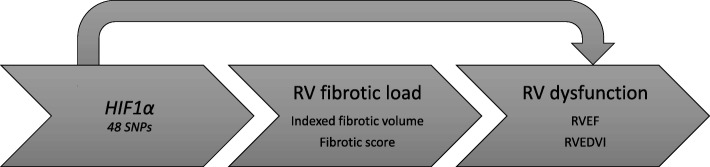
Proposed pathway and associations of interest between *HIF1a*, right ventricular fibrotic load, and right ventricular dysfunction. *HIF1α*, hypoxia inducible factor-1-alpha; RV, right ventricular; RVEDVI, indexed right ventricular end-diastolic volume; RVEF, right ventricular ejection fraction; SNP, single nucleotide polymorphism

We therefore sought to extend our understanding of the relationship between RV fibrosis, RV remodeling, and *HIF1α* genetic variants (Fig. 1).

## Methods

### Study population

Details about this study population have been described [17, 18]. Briefly, patients with TOF were recruited from the Cardiac Center at The Children’s Hospital of Philadelphia (CHOP) from 1992 to 2010 regardless of race or ethnicity [17]. Demographic information (e.g., gender, race/ethnicity) for patients with confirmed diagnosis of TOF was collected via patient or parent interviews. Clinical information including gestational age and details of the cardiac anatomy were ascertained at the time of consent. Additional information including the patient’s height and weight, use of palliative procedure, age at complete repair, type of surgical repair, intervening events since complete repair, pulmonary artery peak velocity, RV outflow tract gradient, and pulmonary regurgitant fraction were abstracted from medical records at the time of the CMR. All cases were screened for 22q11.2 deletion syndrome using fluorescence in situ hybridization and multiplex ligation-dependent probe amplification. Cases determined to carry the 22q11.2 deletion or any other suspected syndromes were excluded from the present study [19]. The Institutional Review Board for the Protection of Human Subjects at CHOP and the Committee for the Protection of Human Subjects at the University of Texas Health Science Center approved this study.

The subset of TOF cases with at least one post-operative CMR at CHOP was selected for this study. Cases had either a research-based or clinically-indicated CMR [18]. Complete repair for TOF was defined as closure of the ventricular septal defect and relief of right-sided outflow tract obstruction. Cases were excluded if they underwent complete repair for TOF after 2 years of age or had their first CMR after 21 years of age (i.e., to exclude those with prolonged exposure to pre-operative hypoxia and pressure overload, or chronic pulmonary regurgitation and post-operative aging). We also excluded cases when the CMR did not allow for the measurement of fibrosis.

### Cardiac magnetic resonance measures

CMRs were performed on at 1.5T (Avanto, Siemens Healthineers, Erlangen, Germany) as previously described [18]. In brief, the protocol included balanced steady-state free-precession cine CMR acquisitions in 4-chamber and long-axis planes and contiguous short-axis cine imaging from base to apex. Thickness of the slices were adjusted to obtain 8–12 slices across the ventricle with in plane resolution ranging from 1.5–2.5 mm. The number of phases ranged from 20 to 30 depending upon the heart rate. Flip angle ranged from 75 to 90 degrees. Parallel imaging was utilized with an acceleration factor of 2. All volumes were indexed to body surface area.

Cardiac fibrosis was measured using an inversion recovery technique for late gadolinium enhancement (LGE). Ten minutes after gadolinium chelate (gado-pentetate dimeglumine) was intravenously administered at a dose of 0.4 ml/kg, imaging was performed with appropriate inversion time to maximize contrast between the normal myocardium and fibrotic areas. Both magnitude and phase sensitive images were reconstructed.

RV fibrosis was quantified using two scales: indexed fibrotic volume and fibrotic score. RV fibrotic volume was calculated by identifying areas of fibrosis (excluding the ventricular septal and transannular patches) and tracing them manually on each slice; the total fibrotic load was quantified by integrating the fibrotic volume on each slice over the entire ventricle. For RV fibrotic score, we used an LGE scoring system, dividing the RV into seven regions, as described by Babu-Narayan et al. [5, 20]. The maximum score was 20. The investigator measuring fibrotic load was blinded to RV function, and the physician reporting on RV function was blinded to fibrotic load measurements.

Phase contrast CMR (PC-CMR) was utilized to determine pulmonary regurgitant fraction. An initial velocity encoding (VENC) of 150 cm/sec was utilized with a flip angle of 25 degrees; if velocities in the pulmonary artery were found to exceed the VENC, a higher VENC was used and the sequence was repeated. Slice thickness was between 4 and 6 mm with the number of phases, similar to cine imaging, a function of the heart rate (between 20 and 30 phases/cardiac cycle).

RV end-diastolic volume indexed (RVEDVI) and RV ejection fraction (RVEF) were measured as previously described [21–24]. The RV infundibulum was included in the RV volume up to the pulmonary annulus. RV end-systolic volume was also measured in the same manner as RV end-diastolic volume to calculate RVEF. RVEF was calculated as:

$$ \mathrm{RVEF}\ \left(\%\right)=\left[\left(\mathrm{RV}\ \mathrm{end}-\mathrm{diastolic}\ \mathrm{volume}-\mathrm{RV}\ \mathrm{end}-\mathrm{systolic}\ \mathrm{volume}\right)/\mathrm{RV}\ \mathrm{end}-\mathrm{diastolic}\ \mathrm{volume}\right]\times 100. $$

Pulmonary regurgitation was measured by planimeterizing the pulmonary artery in cross section on the PC-CMR images and integrating the velocities in each voxel over the vessel across the cardiac cycle; multiplying that stroke volume by the average heart rate during imaging yielded flow. Pulmonary regurgitant fraction was calculated as:

$$ \mathrm{Regurgitant}\ \mathrm{fraction}\ \left(\%\right)=\left(\mathrm{reverse}\ \mathrm{flow}/\mathrm{forward}\ \mathrm{flow}\right)\ \mathrm{x}\ 100. $$

### Genotyping

Existing genotyped and imputed data were available for a subset of patients with TOF, as previously described [17]. Briefly, cases had been genotyped on either the Illumina Infinium™ II HumanHap550K or 610Q BeadChip at CHOP’s Center for Applied Genomics. Genotype data were used to impute additional data based on the 1000 Genomes Project. After applying post-imputation quality control procedures (e.g., excluding single nucleotide polymorphisms [SNPs] with minor allele frequencies < 5%) [17], the 3 SNPs evaluated by Jeewa et al. [16], and 45 additional SNPs were available in the region under study (*HIF1α* gene ±1 kb upstream and downstream).

Cases that had not been previously genotyped were genotyped on the Illumina BeadChip 2.5 M v8 at the same Center for Applied Genomics at CHOP, using DNA extracted from a blood or saliva sample (Puregene DNA isolation kit [Gentra Systems, Inc., Minneapolis, Minnesota, USA] for blood samples, and Oragene DNA isolation kit [DNA Genotek Inc., Ontario, Canada] for saliva samples). For these cases, we repeated the imputation procedures and conducted post-imputation quality control for the *HIF1α* gene ±1 kb upstream and downstream.

### Statistical analysis

We determined counts and frequencies for categorical variables and medians and ranges for continuous variables. For cases ≥ 20 years old, body mass index (BMI) at CMR was calculated as weight (kg) divided by height squared (m^2^). For cases < 20 years old, BMI percentiles at CMR were determined using age and sex-specific growth charts (https://www.cdc.gov/growthcharts/clinical_charts.htm). Cases were categorized into the standard BMI categories. Cases were considered to have residual right-sided obstruction if they had both a main pulmonary artery peak velocity > 2.5 m/s and an RV outflow tract gradient > 30 mmHg on CMR; otherwise, they were considered not to have right-sided obstruction.

To study the association between RV fibrosis (assessed by indexed fibrotic volume and fibrotic score) and RV function (assessed by RVEF and RVEDVI), we used multivariable linear mixed-effects regression with a random intercept. A random intercept was used to account for repeated measurements in cases for whom we had data from two CMRs. We considered the following variables as potential confounders: age at completed surgical repair (0–4 months, 5–8 months, 9–12 months, > 12 months), type of surgical repair (transannular patch, RV to pulmonary artery conduit, nontransannular patch or ventricular septal defect closure only), time between completed surgical repair and CMR (the earliest CMR in patients with results from two CMRs), intervening events (i.e. pulmonary artery intervention, pulmonary valve replacement, revision of RV to branch pulmonary artery conduit, closure of residual septal defect(s), arrhythmias requiring medication or electrophysiologic intervention, use of cardiac medications), BMI at the time of the CMR, the degree of RV outflow tract obstruction at the time of CMR, and pulmonary regurgitation fraction at the time of the CMR [mild (0–20%), moderate (> 20–40%), severe (> 40%)]. To identify covariates to include in the model, first we ran a crude model between RVEF and indexed fibrotic volume. Next, each potential confounder was included in the model and the percent change from the crude estimate was calculated. This process was repeated using fibrotic score as the exposure. Covariates that changed the crude estimate by more than 10% in either model (indexed fibrotic volume or fibrotic score) were considered potential confounders and included in the main analyses. As the time between CMRs varied between cases, we also controlled for time between the two CMRs. Time between CMRs was set to zero for cases with only one CMR. The main analyses were repeated using RVEDVI to assess RV dilation. RVEDVI was log-transformed to meet model assumptions.

We also evaluated RVEF and RVEDVI as dichotomous traits. RVEF < 50% was considered abnormal and RVEF ≥ 50% was normal [25]. For RVEDVI, > 108 cc/m^2^ was considered abnormal and ≤ 108 cc/m^2^ was normal [25]. We used multivariable logistic mixed-effects regression with a random intercept, controlling for the same covariates as included in the multivariable mixed-linear regression. Analyses were performed using SAS version 9.4 (SAS Institute Inc., Cary, North Carolina, USA). For the multivariable models, *p* < 0.05 was considered significant.

### Genetic analyses

We restricted our genetic analyses to cases that identified as being white (to limit the potential for population stratification bias) and had complete covariate data. The small number of non-white cases precluded separate analyses within other race/ethnic groups (*n* < 25 cases). We examined the association between each genetic variant (modeled additively for the minor allele) and each measurement of RV fibrotic load and RV function. Indexed fibrotic volume was square root transformed to meet model assumptions. For continuous outcomes (square root transformed indexed fibrotic volume, RVEF, RVEDVI), we used multivariable linear mixed-effects regression with a random intercept. For fibrotic score, we used a random intercept, multivariable generalized Poisson regression to account for underdispersion. In all models, we controlled for time between completed surgical repair and the first CMR, and time between the first and second CMR. We used a false discovery rate to account for multiple comparisons conducted for each outcome [26, 27]. For all variants, a regional association plot was constructed using LocusZoom [28], using the 1000 Genomes European population (Nov 2014). We also checked for linkage disequilibrium using SNPclip (R^2^ = 0.8, minor allele frequency = 0.01) [29]. All variants were annotated using Combined Annotation Dependent Depletion (CADD) [30] and Genome-Wide Annotation of VAriants (GWAVA) scores [31].

## Results

**Fig. 2:**
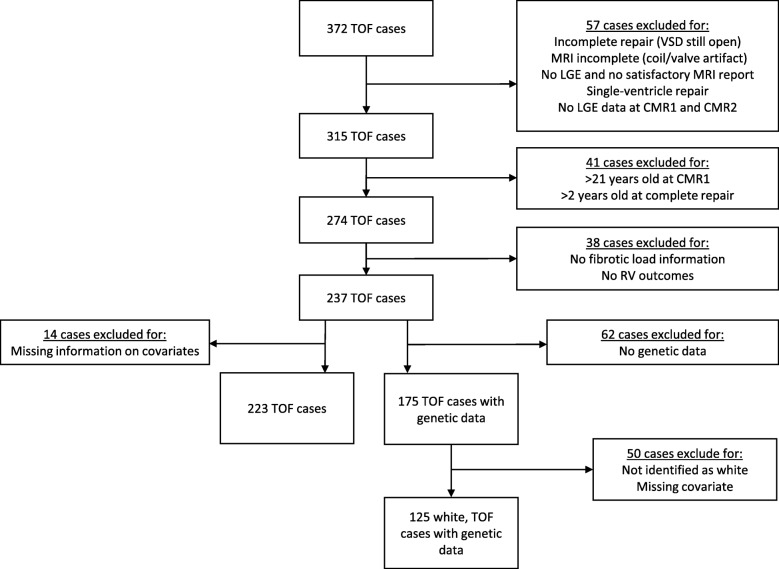
Diagram of study population

A total of 237 cases with repaired TOF were eligible for this study and had at least one measurement of fibrosis (Fig. 2). The distribution of case characteristics at the first and second CMR is presented in Table 1. At the first CMR, patients had a median age of 12.3 years and a median of 11 years had passed since their complete surgical repair. The majority of cases were male (66%), white (78%), and had a normal BMI (53%). At birth, 73% had valvar pulmonary stenosis and most had a complete repair without prior palliation (84%). At the first CMR, most had moderate or severe pulmonary regurgitation (77%). The distribution of most characteristics was similar in cases with a second CMR, but there were more males (74%), more were born premature (27%), and all cases had moderate or severe pulmonary regurgitation.

**Table 1:** Characteristics of study cohort

Characteristic	First CMR (*N* = 237)		Second CMR (*N* = 92)	
	N	%	N	%
Gender				
Male	157	66.2	68	73.9
Female	80	33.8	24	26.1
Race/Ethnicity				
White	182	77.5	68	73.9
African American	23	9.8	10	10.9
Hispanic	13	5.5	5	5.4
Other	17	7.2	9	9.8
Missing	2	–	0	–
Body Mass Index (kg/m^2^)				
Underweight (< 18.5)	51	21.5	23	25.0
Normal (18.5 to < 25)	125	52.7	51	55.4
Overweight (25 to < 30)	34	14.4	9	9.8
Obese (≥30)	27	11.4	9	9.8
Prematurity				
Yes	40	18.4	22	26.8
No	177	81.6	60	73.2
Missing	20	–	10	–
Pulmonary Valve Anatomy				
Absent	18	7.9	7	7.8
Atresia	43	18.8	20	22.2
Stenosis	168	73.4	63	70.0
Missing	8	–	2	–
RVOT Obstruction				
Yes	18	8.1	10	11.0
No	203	91.9	81	89.0
Missing	16	–	1	–
Palliative Procedure				
Yes	37	15.6	18	19.6
No	200	84.4	74	80.4
Age at complete repair (months)				
0–4	149	63.1	52	56.5
5–8	49	20.8	20	21.7
9–12	22	9.3	11	12.0
> 12	16	6.8	9	9.8
Missing	1	–	0	–
Type of Surgical Repair				
Transannular Patch	176	75.2	71	77.2
Right Ventricle to Pulmonary Artery Conduit	35	15.0	13	14.1
Nontransannular Patch or Ventricular Septal Defect Closure Only	23	9.8	8	8.7
Missing	3	–	0	–
Intervening events since complete repair				
Yes	120	50.6	56	60.9
No	117	49.4	36	39.1
Pulmonary Artery Confluence				
Yes	217	93.1	87	95.6
No	16	6.9	4	4.4
Missing	14	–	1	–
Pulmonary Regurgitant Fraction				
Mild (0–20)	53	23.0	0	0
Moderate (> 20–40)	90	39.1	37	50.0
Severe (> 40)	87	37.8	37	50.0
Missing	7	–	18	–
	Median	IQR	Median	IQR
Age (years)	12.3	8.7–16.2	15.8	12.2–18.7
Time between complete repair and first CMR (years)	11.0	8–15	15.0	11–18
RV fibrotic volume, indexed (ml/m^2^)	0.6	0.3–1.0	0.7	0.4–1.0
RV fibrotic score	2	1–3	2	1–3
RVEDVI	112.0	89.7–140.0	125.9	105.5–144.6
RVEF (%)	58.8	52.8–63.8	56.6	50.5–60.9

*CMR* cardiovascular magnetic resonance, *IQR* interquartile range, *RV* right ventricular, *RVEDVI* indexed right ventricular end-diastolic volume, *RVEF* right ventricular ejection fraction, *RVOT* right ventricular outflow tract

### RV fibrotic load and RV function

After adjusting for time between completed surgical repair and first CMR, BMI, obstruction, and pulmonary regurgitant fraction, neither indexed fibrotic volume nor fibrotic score were associated with log-transformed RVEDVI. However, both were significantly associated with RVEF (modeled continuously) (Table 2). RVEF decreased by 1.6% (*p* = 0.04), for every one-unit increase in indexed fibrotic volume and decreased by 0.9% (*p* = 0.03) for every one-unit increase in fibrotic score.

**Table 2:** Relationship between right ventricular fibrotic load and right ventricular function among cases with repaired tetralogy of Fallot

	Indexed fibrotic volume			Fibrotic score		
Linear Model	β^a^	SE	*p*-value	β^a^	SE	*p*-value
RVEF	−1.58	0.78	0.04	−0.89	0.39	0.03
Log RVEDVI	0.03	0.02	0.25	0.02	0.01	0.18
Dichotomized model^b^	OR^a^	95% CI	*p*-value	OR^a^	95% CI	*p*-value
RVEF	1.72	0.99, 2.96	0.052	1.37	1.06, 1.78	0.02
RVEDVI	1.21	0.72, 2.03	0.46	1.06	0.81, 1.38	0.68

*CI* confidence interval, *RVEDVI* indexed right ventricular end-diastolic volume, *RVEF* right ventricular ejection fraction, *OR* odds ratio, *SE* standard error

^a^ Adjusted for time between the first and second CMR, time between repair and first CMR, body mass index, pulmonary regurgitant fraction, and obstruction

^b^ RVEF< 50%, RVEDVI> 108 cc/m^2^ considered abnormal

When RV volume and function were analyzed as dichotomous variables (RVEDVI > 108 cc/m^2^ and RVEF < 50% were considered abnormal, respectively), RVEDVI was not significantly associated with indexed fibrotic volume (OR = 1.21, 95% CI: 0.72, 2.03; *p* = 0.46) or fibrotic score (OR = 1.06, 95% CI: 0.81, 1.38; *p* = 0.68) (Table 2). However, the odds of having an abnormal RVEF increased by 72% for every one-unit increase in indexed fibrotic volume (95% CI: 0.99, 2.96; *p* = 0.05) and by 37% (95% CI: 1.06, 1.78; *p* = 0.02) for every one-unit increase in fibrotic score.

### HIF1α, RV function, and RV fibrotic load

A total of 125 white cases had genetic data and complete information on covariates (Fig. 2). In this subset, the distribution of case characteristics at the first and second CMR is similar to the distribution reported in Table 1, except the time between complete repair and first CMR is lower at the second CMR (median of 10 years in subset vs 15 years in study population) (data not shown). None of the 48 *HIFIα* variants were associated with either RVEF or RVEDVI (Additional file 1: Table S1).

**Fig. 3:**
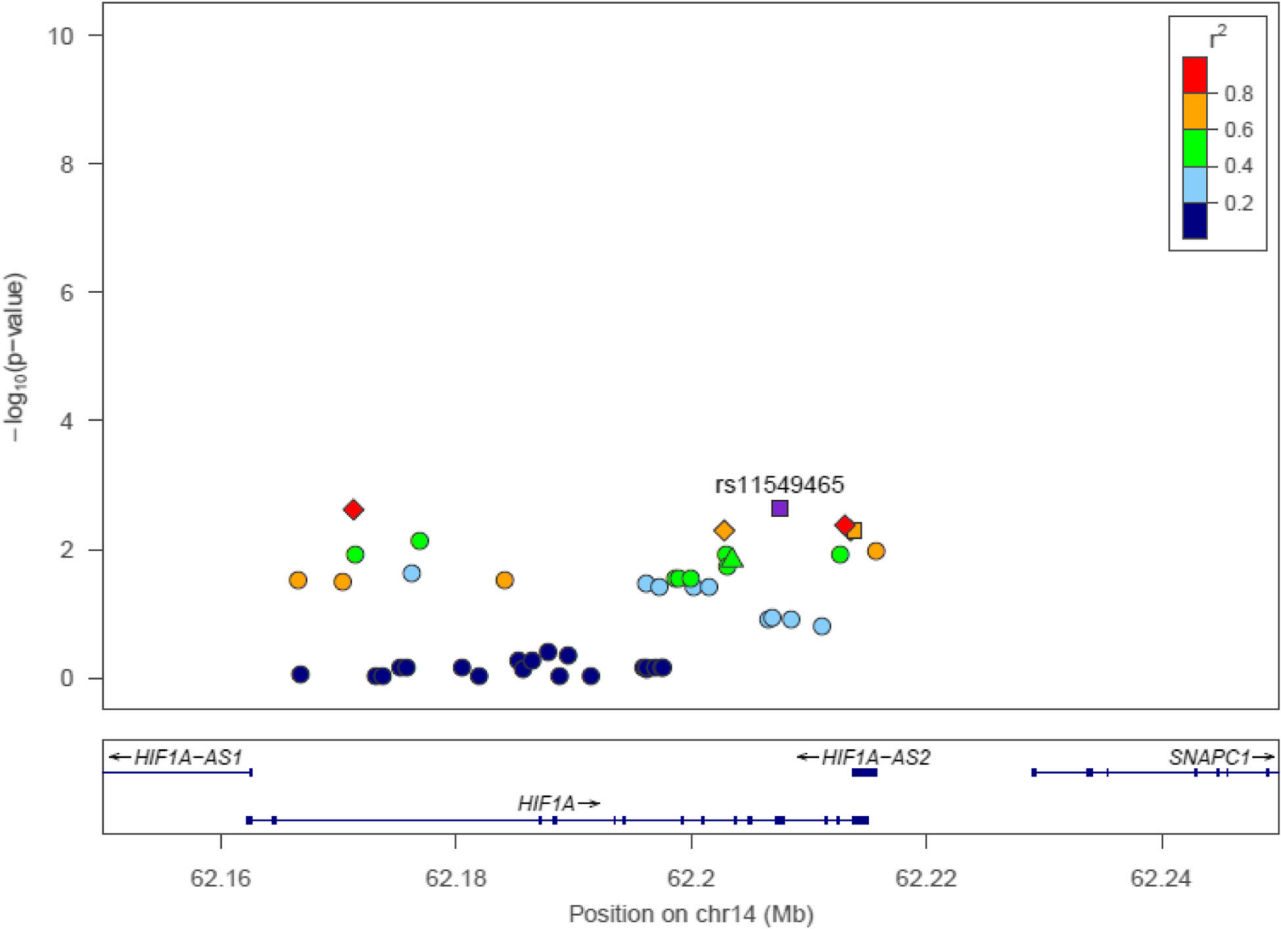
Regional association plot constructed from LocusZoom for the 48 *HIF1α* variants, based on the 1000 Genomes European population (Nov 2014). Each point represents one of the 48 *HIF1α* variants. The r^2^ represents the linkage disequilibrium (i.e., correlation) between each variant and rs11549465. For example, the linkage disequilibrium between the two variants shaded in red and rs11549465 ranges from 0.80 to 1.0. These three variants reflect a unique signal. The linkage disequilibrium between variants shaded in yellow and rs11549465 ranges from 0.60 to 0.80. The variants shaded in yellow reflect another unique signal. Square: variants that were in Jeewa et al.’s study and significant in our study (purple square = rs11549465, yellow square = rs2057482); Triangle: variant analyzed in Jeewa et al.’s study but not significant in our study (green triangle = rs10873142); Diamond: variants significant in this study (left red diamond = rs76308410, yellow diamond = rs7161527, right red diamond = rs74481028, yellow diamond (underneath rs74481028): rs10147275; Circles: variants analyzed in our study but were not significant

After accounting for multiple comparisons, none of the variants were significantly associated with square root transformed fibrotic volume (Additional file 1: Table S2). However, six *HIF1α* SNPs (i.e., rs76308410, rs11549465, rs74481028, rs7161527, rs10147275, and rs2057482) were significantly (FDR *p* < 0.05) associated with fibrotic score (Table 3). (The full results for the association between fibrotic score and each of the 48 variants are available in Additional file 1: Table S2). Based on LDlink, these six SNPs are in linkage disequilibrium and represent two unique signals. Rs76308410, rs11549465, and rs74481028 are in linkage disequilibrium with each other (i.e., highly correlated). Likewise, rs7161527, rs10147275, and rs2057482 are in linkage disequilibrium with each other. However, correlations between SNPs in the two different groups (e.g., rs76308410 and rs7161527) are low (r^2^ < 0.80). Fig. 3 illustrates the linkage disequilibrium across all 48 SNPs.

**Table 3:** Significant associations between SNPs within *HIF1α* and fibrotic score among white cases with repaired tetralogy of Fallot

SNP^a^	Position^b^	Risk allele	Function	Fibrotic score		N	CADD score^e^	GWAVA TSS score^f^
				*p*-value^c^	RR (95% CI)^d^			
rs76308410 rs11549465 rs74481028	62,171,263 62,207,557 62,213,060	T T G	Intronic Missense Intronic	0.04 0.04 0.04	1.43 (1.14, 1.79) 1.43 (1.14, 1.78) 1.37 (1.11, 1.70)	124 125 125	11.49 21.20 0.12	0.17 0.31 0.13
rs7161527 rs10147275 rs2057482	62,202,799 62,213,553 62,213,848	T T T	Intronic Intronic Regulatory	0.04 0.04 0.04	1.33 (1.09, 1.62) 1.33 (1.09, 1.62) 1.33 (1.09, 1.62)	125 125 125	1.58 0.01 8.57	0.11 0.15 0.57

*HIF1α* hypoxia inducible factor-1-alpha, *RR* relative risk, *SE* standard error, *SNP* single nucleotide polymorphism, *TSS* transcription start site

^a^ The line demarcates variants that are in linkage disequilibrium (i.e., highly correlated) based on LDlink’s SNPclip (R^2^ = 0.8, MAF = 0.01). The top three SNPs are at least 80% correlated and bottom three SNPs are at least 80% correlated. The full results for the association between fibrotic score and each of the 48 variants are available in Additional file 1: Table S2

^b^ Position is based on the information from Genome Reference Consortium Human Build 37 (GRCh37) (also known as hg19)

^c^ *P*-values are adjusted using false discovery rate to account for multiple testing

^d^ Adjusted for time between surgical repair and the first CMR, time between the first and second CMR

^e^ Scaled Combined Annotation Dependent Depletion (CADD) - variants with CADD >10 are predicted to fall in the top 10% of the most deleterious variants in the genome

^f^ Genome-Wide Annotation of Variants (GWAVA) score - predicts the functional impact of non-coding variants/regions (range 0–1)

## Discussion

Our results support an inverse association between RV fibrotic load and RVEF on CMR in patients with repaired TOF. Similar results have been reported in most studies investigating this association [5, 8, 32, 33]. Previous reports on RV fibrotic load and RVEDVI have been inconsistent [5, 33]. We did not find evidence of an association. These results suggest that RV fibrosis modifies RV function, though its impact on RV remodeling is not fully understood.

We also found that six *HIF1α* SNPs were significantly associated with fibrotic score but not fibrotic volume. While both are seemingly valid measures of fibrotic burden, we speculate that the fibrotic score may better reflect the global myocardial response to a diffuse process such as hypoxia, while the fibrotic volume may reflect the individual response to local injury from suturing or wall stress.

Jeewa et al. were the first to report an association between *HIF1α* variants and RV fibrosis and remodeling [16]. In their study of three *HIF1α* variants, they found evidence that individuals carrying more of the common alleles had more fibrosis at the time of initial surgery, as compared to those with fewer common alleles. However, the reciprocal association was observed for post-surgical progression: The minor alleles at these three variants were associated with more progression (less favorable RV phenotypes) than were the common alleles. Similarly, in our post-surgical TOF cases, the minor alleles at multiple *HIF1α* variants, including the three variants studied by Jeewa et al., were associated with higher fibrotic scores as compared to the more common alleles: rs10873142 RR = 1.26 (1.05, 1.52) [Additional file 1: Table S2]; rs2057482 RR = 1.33 (1.09, 1.62); rs11549465 RR = 1.43 (1.14, 1.78). Although the specific timing and outcomes evaluated by our group and Jeewa et al. differ, the results from both studies suggest that individuals carrying the minor alleles for several *HIF1α* variants may have poorer outcomes post-surgery than those carrying the more common alleles.

With little regenerative capacity, cardiac injury initiates a process whereby cardiac fibroblasts are stimulated to become myofibroblasts, which secrete elevated levels of collagen and other extracellular matrix proteins. While initially providing structural support, the accumulation of myocardial fibrosis and scar leads to pathologic remodeling and dysfunction [15]. Hypoxia, as well as pressure and volume overload, have been shown to initiate this process, though the mechanisms and origin of cardiac fibroblasts remain poorly defined. Patients with TOF are exposed to each of these physiologic stresses over time and have been found to harbor both RV and left ventricular fibrosis pre- and post-operatively [9, 34]. Our results suggest that the hypoxia response pathway contributes to the development of fibrosis, and that genetic variation may modulate individual response. However, given the variety of exposures, the hypoxia response pathway (and *HIF1α* in particular) is likely only one mechanism by which TOF patients develop fibrosis. Identification of the genes, pathways, and variants that modulate this process may help identify patients who are genetically at risk for a greater fibrotic response to physiologic stresses.

Understanding modulators of fibrosis may create opportunities to intervene with anti-fibrotic agents. Several trials have already attempted to use anti-fibrotic agents to modify outcomes in related disease states. For example, aldosterone stimulates receptors in the cardiomyocytes and fibroblasts, which induces myocardial fibrosis. Studies have tested whether aldosterone antagonists (e.g., eplerenone) can reverse myocardial fibrosis or alter surrogates of myocardial fibrosis (e.g., serum markers of collagen turnover). In a clinical trial of adults with D-transposition of the great arteries and systemic RV (after the atrial switch operation), eplerenone resulted in significant changes in serum levels of collagen turnover biomarkers, although its use after 1 year was not associated with changes in RV mass and function. In adults, studies targeting the pro-fibrotic renin-angiotensin-aldosterone system have shown that spironolactone, losartan, and others have resulted in significant reductions in the collagen volume fraction measured histologically [35, 36]. Another study utilizing spironolactone in heart failure patients with preserved ejection fraction (associated with myocardial fibrosis) showed an improvement in exercise and diastolic function [37], while another in cardiomyopathy patients demonstrated reduced ventricular tachycardia [38]. Thus, identifying and treating the TOF patient at risk for ventricular fibrosis might curtail pathologic RV remodeling and improve long-term outcomes.

The interplay between genotype, environment (i.e., exposures), management decisions, and outcomes is complicated and remains unanswered by this single retrospective study. While our study suggests an association between *HIF1α* variants and fibrosis while controlling for age of surgery and thus length of exposure to hypoxia, it cannot address the complex equation whether earlier versus later intervention with longer or shorter exposure to hypoxia is preferred based on genotype. As noted, there may be additional genetic modifiers of the fibrotic response to multiple stimuli encountered along a patient’s clinical course, and there are many other known and unknown factors that contribute to clinical outcomes. As such, only a well-powered, prospective study that assesses genotype, disease severity, and prospective changes in biventricular fibrosis, remodeling, and function can definitively provide individually tailored management strategies.

### Limitations

Given the absence of T1 images on the available CMRs, we could not measure diffuse fibrosis. Instead, we used LGE, which is known to correlate well with scar tissue and provides a good estimate of focal or reactive fibrosis [39]. We included surgical approach to account for the potential extent of surgical disruption of RV myocardium, but we were unable to detail the size of surgical incisions or quantify the extent of muscle bundle resection. Most fibrosis is presumably secondary to surgical disruption of the ventricular myocardium, but we and others have observed fibrosis outside of surgical sites including the RV inferior and anterior walls, and RV septal surface and septal insertion points [5]. Because a single investigator measured fibrosis independent of RV function, biases in measuring fibrosis were probably minimal. Given that some CMRs were performed for clinical purposes, the study population might be biased toward those with more severe clinical circumstances or cardiac status. However, many patients in our practice undergo routine surveillance by CMR and the study cohort displayed a broad range of RV characteristics and fibrotic load, suggesting that the disease-spectrum was somewhat representative of a general TOF population. Our genetic analyses had a small sample size, but we included repeated CMR measures to improve power. We only studied variants from one gene, but the relationship is probably genetically more complex than SNPs within a single gene.

## Conclusions

In patients with repaired TOF, our results suggest there is an inverse association between RV fibrotic load and RVEF on CMR. Our results also suggest certain minor alleles in *HIF1α* are associated with RV fibrosis but not RVEF nor RVEDVI in patients with repaired TOF. While additional studies must be performed to validate our observations, these findings support further study of the hypoxia-response pathway and pathways contributing to ventricular fibrosis. Identifying genotypes associated with increased fibrosis may allow the selection of at-risk patients for novel anti-fibrotic therapies to obviate deleterious RV remodeling and improve long-term clinical outcomes.

## Additional file

Additional file 1: **Table S1.** Associations between SNPs within *HIF1α* and RV function among white repaired tetralogy of Fallot cases. **Table S2.** Associations between SNPs within *HIF1α* and fibrotic load among white cases with repaired tetralogy of Fallot. (DOCX 44 kb)

## Data Availability

The datasets generated and/or analyzed during the current study are not publicly available but are available from the corresponding author on reasonable request.

## Abbreviations

BMI: Body mass index
CADD: Combined annotation dependent depletion
CHOP: The Children’s Hospital of Philadelphia
CMR: Cardiovascular magnetic resonance
GWAVA: Genome-wide annotation of variants
*HIF1α*: Hypoxia inducible factor-1-alpha
IQR: Interquartile range
LGE: Late gadolinium enhancement
PC-CMR: Phased contrast cardiovascular magnetic resonance
RV: Right ventricle/right ventricular
RVEDVI: Right ventricular end-diastolic volume index
RVEF: Right ventricular ejection fraction
SNPs: Single nucleotide polymorphisms
*TGFβ1*: Transforming growth factor β1
TOF: Tetralogy of Fallot
VENC: Velocity encoding

## References

[1] Shuler CO, Black GB, Jerrell JM (2013). Population-based treated prevalence of congenital heart disease in a pediatric cohort. Pediatr Cardiol.

[2] Hickey EJ, Veldtman G, Bradley TJ, Gengsakul A, Manlhiot C, Williams WG, Webb GD, McCrindle BW (2009). Late risk of outcomes for adults with repaired tetralogy of Fallot from an inception cohort spanning four decades. Eur J Cardiothorac Surg.

[3] Marelli AJ, Ionescu-Ittu R, Mackie AS, Guo L, Dendukuri N, Kaouache M (2014). Lifetime prevalence of congenital heart disease in the general population from 2000 to 2010. Circulation.

[4] Bouzas B, Kilner PJ, Gatzoulis MA (2005). Pulmonary regurgitation: not a benign lesion. Eur Heart J.

[5] Babu-Narayan SV, Kilner PJ, Li W, Moon JC, Goktekin O, Davlouros PA, Khan M, Ho SY, Pennell DJ, Gatzoulis MA (2006). Ventricular fibrosis suggested by cardiovascular magnetic resonance in adults with repaired tetralogy of fallot and its relationship to adverse markers of clinical outcome. Circulation.

[6] Deanfield JE, Ho SY, Anderson RH, McKenna WJ, Allwork SP, Hallidie-Smith KA (1983). Late sudden death after repair of tetralogy of Fallot: a clinicopathologic study. Circulation.

[7] Moore JP, Seki A, Shannon KM, Mandapati R, Tung R, Fishbein MC (2013). Characterization of anatomic ventricular tachycardia isthmus pathology after surgical repair of tetralogy of Fallot. Circ Arrhythm Electrophysiol.

[8] Munkhammar P, Carlsson M, Arheden H, Pesonen E (2013). Restrictive right ventricular physiology after tetralogy of Fallot repair is associated with fibrosis of the right ventricular outflow tract visualized on cardiac magnetic resonance imaging. Eur Heart J Cardiovasc Imaging.

[9] Chen CA, Dusenbery SM, Valente AM, Powell AJ, Geva T (2016). Myocardial ECV fraction assessed by CMR is associated with type of hemodynamic load and arrhythmia in repaired tetralogy of Fallot. JACC Cardiovasc Imaging.

[10] Riesenkampff E, Luining W, Seed M, Chungsomprasong P, Manlhiot C, Elders B, McCrindle BW, Yoo SJ, Grosse-Wortmann L (2016). Increased left ventricular myocardial extracellular volume is associated with longer cardiopulmonary bypass times, biventricular enlargement and reduced exercise tolerance in children after repair of tetralogy of Fallot. J Cardiovasc Magn Reson.

[11] Wald RM, Haber I, Wald R, Valente AM, Powell AJ, Geva T (2009). Effects of regional dysfunction and late gadolinium enhancement on global right ventricular function and exercise capacity in patients with repaired tetralogy of Fallot. Circulation.

[12] Yim D, Riesenkampff E, Caro-Dominguez P, Yoo SJ, Seed M, Grosse-Wortmann L (2017). Assessment of diffuse ventricular myocardial fibrosis using native T1 in children with repaired tetralogy of Fallot. Circ Cardiovasc Imaging.

[13] Zeisberg EM, Tarnavski O, Zeisberg M, Dorfman AL, McMullen JR, Gustafsson E, Chandraker A, Yuan X, Pu WT, Roberts AB (2007). Endothelial-to-mesenchymal transition contributes to cardiac fibrosis. Nat Med.

[14] Sun S, Ning X, Zhang Y, Lu Y, Nie Y, Han S, Liu L, Du R, Xia L, He L (2009). Hypoxia-inducible factor-1alpha induces twist expression in tubular epithelial cells subjected to hypoxia, leading to epithelial-to-mesenchymal transition. Kidney Int.

[15] Travers JG, Kamal FA, Robbins J, Yutzey KE, Blaxall BC (2016). Cardiac fibrosis: the fibroblast awakens. Circ Res.

[16] Jeewa A, Manickaraj AK, Mertens L, Manlhiot C, Kinnear C, Mondal T, Smythe J, Rosenberg H, Lougheed J, McCrindle BW (2012). Genetic determinants of right-ventricular remodeling after tetralogy of Fallot repair. Pediatr Res.

[17] Agopian AJ, Mitchell LE, Glessner J, Bhalla AD, Sewda A, Hakonarson H, Goldmuntz E (2014). Genome-wide association study of maternal and inherited loci for conotruncal heart defects. PLoS One.

[18] Mercer-Rosa L, Paridon SM, Fogel MA, Rychik J, Tanel RE, Zhao H, Zhang X, Yang W, Shults J, Goldmuntz E (2015). 22q11.2 deletion status and disease burden in children and adolescents with tetralogy of Fallot. Circ Cardiovasc Genet.

[19] Agopian AJ, Goldmuntz E, Hakonarson H, Sewda A, Taylor D, Mitchell LE (2017). Pediatric cardiac genomics C: genome-wide association studies and meta-analyses for congenital heart defects. Circ Cardiovasc Genet.

[20] Cerqueira MD, Weissman NJ, Dilsizian V, Jacobs AK, Kaul S, Laskey WK, Pennell DJ, Rumberger JA, Ryan T, Verani MS (2002). Standardized myocardial segmentation and nomenclature for tomographic imaging of the heart. A statement for healthcare professionals from the cardiac imaging Committee of the Council on Clinical Cardiology of the American Heart Association. Circulation.

[21] Chan SS, Whitehead KK, Kim TS, Fu GL, Keller MS, Fogel MA, Harris MA (2015). Repaired tetralogy of Fallot with coexisting unrepaired partial anomalous pulmonary venous connection is associated with diminished right ventricular ejection fraction and more severe right ventricular dilation. Pediatr Radiol.

[22] Fogel MA, Pawlowski T, Keller MS, Cohen MS, Goldmuntz E, Diaz L, Li C, Whitehead KK, Harris MA (2015). The cardiovascular effects of obesity on ventricular function and mass in patients after tetralogy of Fallot repair. J Pediatr.

[23] Mercer-Rosa L, Ingall E, Zhang X, McBride M, Kawut S, Fogel M, Paridon S, Goldmuntz E (2015). The impact of pulmonary insufficiency on the right ventricle: a comparison of isolated valvar pulmonary stenosis and tetralogy of fallot. Pediatr Cardiol.

[24] Whitehead KK, Harris MA, Glatz AC, Gillespie MJ, DiMaria MV, Harrison NE, Dori Y, Keller MS, Rome JJ, Fogel MA (2015). Status of systemic to pulmonary arterial collateral flow after the fontan procedure. Am J Cardiol.

[25] Kawel-Boehm N, Maceira A, Valsangiacomo-Buechel ER, Vogel-Claussen J, Turkbey EB, Williams R, Plein S, Tee M, Eng J, Bluemke DA (2015). Normal values for cardiovascular magnetic resonance in adults and children. J Cardiovasc Magn Reson.

[26] Benjamini Y, Hochberg Y (1995). Controlling the false discovery rate: a practical and powerful approach to multiple testing. J R Stat Soc Series B (Methodological).

[27] Anderson ML (2008). Multiple inference and gender differences in the effects of early intervention: a reevaluation of the abecedarian, Perry preschool, and early training projects. J Am Stat Assoc.

[28] Pruim RJ, Welch RP, Sanna S, Teslovich TM, Chines PS, Gliedt TP, Boehnke M, Abecasis GR, Willer CJ (2010). LocusZoom: regional visualization of genome-wide association scan results. Bioinformatics.

[29] Machiela MJ, Chanock SJ (2015). LDlink: a web-based application for exploring population-specific haplotype structure and linking correlated alleles of possible functional variants. Bioinformatics.

[30] Kircher M, Witten DM, Jain P, O'Roak BJ, Cooper GM, Shendure J (2014). A general framework for estimating the relative pathogenicity of human genetic variants. Nat Genet.

[31] Ritchie GR, Dunham I, Zeggini E, Flicek P (2014). Functional annotation of noncoding sequence variants. Nat Methods.

[32] Dobson RJ, Mordi I, Danton MH, Walker NL, Walker HA, Tzemos N (2017). Late gadolinium enhancement and adverse outcomes in a contemporary cohort of adult survivors of tetralogy of Fallot. Congenit Heart Dis.

[33] Kozak MF, Redington A, Yoo SJ, Seed M, Greiser A, Grosse-Wortmann L (2014). Diffuse myocardial fibrosis following tetralogy of Fallot repair: a T1 mapping cardiac magnetic resonance study. Pediatr Radiol.

[34] Broberg CS, Huang J, Hogberg I, McLarry J, Woods P, Burchill LJ, Pantely GA, Sahn DJ, Jerosch-Herold M (2016). Diffuse LV myocardial fibrosis and its clinical associations in adults with repaired tetralogy of Fallot. JACC Cardiovasc Imaging.

[35] Diez J, Querejeta R, Lopez B, Gonzalez A, Larman M, Martinez Ubago JL (2002). Losartan-dependent regression of myocardial fibrosis is associated with reduction of left ventricular chamber stiffness in hypertensive patients. Circulation.

[36] Izawa H, Murohara T, Nagata K, Isobe S, Asano H, Amano T, Ichihara S, Kato T, Ohshima S, Murase Y (2005). Mineralocorticoid receptor antagonism ameliorates left ventricular diastolic dysfunction and myocardial fibrosis in mildly symptomatic patients with idiopathic dilated cardiomyopathy: a pilot study. Circulation.

[37] Kosmala W, Rojek A, Przewlocka-Kosmala M, Wright L, Mysiak A, Marwick TH (2016). Effect of aldosterone antagonism on exercise tolerance in heart failure with preserved ejection fraction. J Am Coll Cardiol.

[38] Dimas V, Ayers C, Daniels J, Joglar JA, Hill JA, Naseem RH (2011). Spironolactone therapy is associated with reduced ventricular tachycardia rate in patients with cardiomyopathy. Pacing Clin Electrophysiol.

[39] Rathod RH, Powell AJ, Geva T (2016). Myocardial fibrosis in congenital heart disease. Circ J.

