# Supplementary material for: Genetic variants of HIF1α are associated with right ventricular fibrotic load in repaired tetralogy of Fallot patients: a cardiovascular magnetic resonance study
Source: J Cardiovasc Magn Reson. 2019 Aug 19;21:51. doi: 10.1186/s12968-019-0555-2 (PMC6699069; doi:10.1186/s12968-019-0555-2)
Supplement: Supplementary file 1 — Table S1. Associations between SNPs within HIF1α and RV function among white repaired tetralogy of Fallot cases. Table S2. Associations between SNPs within HIF1α and fibrotic load among white cases with repaired tetralogy of Fallot. (DOCX 44 kb) [file 12968_2019_555_MOESM1_ESM.docx]

**Supplementary Table 1** Associations between SNPs^a^ within *HIF1α* and RV function among white repaired tetralogy of Fallot cases

| SNP | Position^b^ | Risk allele | Function | Square root fibrotic volume | | | Fibrotic score | | N | CADD score ^e^ | GWAVA TSS score^f^ |
| --- | --- | --- | --- | --- | --- | --- | --- | --- | --- | --- | --- |
|  |  |  |  | p-value^c^ | | β (SE)^d^ | p-value^c^ | RR (95% CI)^d^ |  |  |  |
| rs2301106 | 62166563 | C | Intergenic | 0.99 | 0.47 (1.28) | | 0.60 | -2.95 (5.41) | 125 | 17.57 | 0.39 |
| rs78703581 | 62184128 | A | Transcript | 0.99 | 0.47 (1.28) | | 0.60 | -2.95 (5.41) | 125 | 3.84 | 0.24 |
| rs11851112 | 62170340 | T | Transcript | 0.99 | 0.66 (1.29) | | 0.60 | -2.98 (5.46) | 124 | 0.02 | 0.20 |
| rs6573399 | 62215725 | T | Intergenic | 0.99 | -0.38 (1.30) | | 0.60 | -3.57 (5.69) | 104 | 4.06 | 0.14 |
| rs76308410 | 62171263 | T | Intronic | 0.99 | -0.35 (1.67) | | 0.59 | -5.28 (7.16) | 124 | 11.49 | 0.17 |
| rs11549465 | 62207557 | T | Missense | 0.99 | -0.69 (1.65) | | 0.59 | -5.15 (7.03) | 125 | 21.20 | 0.31 |
| rs74481028 | 62213060 | G | Intronic | 0.99 | -1.06 (1.56) | | 0.54 | -6.44 (6.57) | 125 | 0.12 | 0.13 |
| rs7161527 | 62202799 | T | Intronic | 0.99 | -0.88 (1.41) | | 0.54 | -5.12 (5.99) | 125 | 1.58 | 0.11 |
| rs10147275 | 62213553 | T | Intronic | 0.99 | -0.88 (1.41) | | 0.54 | -5.12 (5.99) | 125 | 0.01 | 0.15 |
| rs2057482 | 62213848 | T | Regulatory | 0.99 | -0.88 (1.41) | | 0.54 | -5.12 (5.99) | 125 | 8.57 | 0.57 |
| rs1951795 | 62171426 | A | Transcript | 0.99 | -0.81 (1.24) | | 0.54 | -6.30 (5.22) | 125 | 12.38 | 0.27 |
| rs12435848 | 62176891 | G | Intergenic | 0.99 | 0.01 (1.09) | | 0.54 | -4.81 (4.60) | 125 | 2.65 | 0.24 |
| rs12891737 | 62196186 | C | Intergenic | 0.99 | -0.06 (1.13) | | 0.54 | -4.47 (4.67) | 119 | 3.09 | 0.03 |
| rs12435848 | 62176891 | A | Transcript | 0.99 | -0.31 (1.22) | | 0.54 | -7.47 (5.17) | 124 | 2.71 | 0.24 |
| rs2301111 | 62200201 | G | Intergenic | 0.99 | 0.01 (1.09) | | 0.54 | -4.81 (4.60) | 125 | 7.99 | 0.13 |
| rs75606298 | 62201500 | G | Transcript | 0.99 | 0.01 (1.09) | | 0.54 | -4.81 (4.60) | 125 | 6.22 | 0.11 |
| rs4899057 | 62202942 | G | Transcript | 0.99 | -0.81 (1.24) | | 0.54 | -6.30 (5.22) | 125 | 0.10 | 0.06 |
| rs10873142 | 62203462 | C | Transcript | 0.99 | -1.13 (1.24) | | 0.54 | -5.72 (5.29) | 123 | 2.63 | 0.10 |
| rs4902082 | 62212675 | C | Transcript | 0.99 | -0.81 (1.24) | | 0.54 | -6.30 (5.22) | 125 | 5.05 | 0.23 |
| rs11621525 | 62203056 | A | Transcript | 0.99 | -0.61 (1.25) | | 0.54 | -6.45 (5.58) | 105 | 0.64 | 0.06 |
| rs2301113 | 62206548 | C | Transcript | 0.99 | -1.14 (1.16) | | 0.60 | -2.93 (5.33) | 96 | 2.18 | 0.07 |
| rs2301115 | 62206913 | T | Transcript | 0.99 | -1.09 (1.17) | | 0.59 | -3.57 (5.30) | 95 | 2.28 | 0.04 |
| rs4902081 | 62208474 | C | Transcript | 0.99 | -1.14 (1.16) | | 0.60 | -2.93 (5.33) | 96 | 1.23 | 0.04 |
| rs10135579 | 62173187 | G | Transcript | 0.99 | -0.53 (2.44) | | 0.54 | -9.06 (10.20) | 125 | 3.36 | 0.26 |
| rs76276969 | 62173727 | C | Transcript | 0.99 | -0.53 (2.44) | | 0.54 | -9.06 (10.20) | 125 | 0.60 | 0.23 |
| rs10129270 | 62181954 | A | Regulatory | 0.99 | -1.23 (2.14) | | 0.54 | -10.17 (8.78) | 125 | 3.97 | 0.46 |
| rs77830262 | 62188791 | A | Transcript | 0.99 | -0.53 (2.44) | | 0.54 | -9.06 (10.20) | 125 | 2.68 | 0.20 |
| rs10144011 | 62191482 | T | Transcript | 0.99 | -0.53 (2.44) | | 0.54 | -9.06 (10.20) | 125 | 13.70 | 0.22 |
|  |  |  |  |  |  | |  |  |  |  |  |
|  |  |  |  |  |  | |  |  |  |  |  |
|  |  |  |  |  |  | |  |  |  |  |  |
| rs12882000 | 62175273 | T | Transcript | 0.99 | -0.84 (1.75) | | 0.54 | -6.70 (7.31) | 125 | 11.47 | 0.17 |
| rs12881961 | 62175801 | G | Transcript | 0.99 | -0.84 (1.75) | | 0.54 | -6.70 (7.31) | 125 | 0.30 | 0.15 |
| rs10459450 | 62180485 | A | Intergenic | 0.99 | -0.84 (1.75) | | 0.54 | -6.70 (7.31) | 125 | 9.58 | 0.20 |
| rs4899056 | 62189531 | T | Transcript | 0.99 | 0.50 (1.67) | | 0.54 | -7.32 (6.95) | 125 | 6.65 | 0.14 |
| rs12887165 | 62195945 | C | Intergenic | 0.99 | -0.84 (1.75) | | 0.54 | -6.70 (7.31) | 125 | 1.52 | 0.00 |
| rs71423444 | 62196197 | C | Intergenic | 0.99 | -1.29 (1.81) | | 0.54 | -6.67 (7.47) | 118 | 1.64 | 0.07 |
| rs67229007 | 62196204 | A | Intergenic | 0.99 | -0.84 (1.75) | | 0.54 | -6.70 (7.31) | 125 | 0.47 | 0.07 |
| rs1957757 | 62196948 | T | Intergenic | 0.99 | -0.84 (1.75) | | 0.54 | -6.70 (7.31) | 125 | 3.70 | 0.08 |
| rs8005745 | 62185295 | T | Transcript | 0.99 | 0.03 (1.68) | | 0.54 | -8.89 (7.04) | 124 | 3.24 | 0.17 |
| rs2284999 | 62186478 | C | Transcript | 0.99 | 0.03 (1.68) | | 0.54 | -8.89 (7.04) | 124 | 13.51 | 0.19 |
| rs2301108 | 62197464 | A | Intergenic | 0.99 | -0.84 (1.75) | | 0.54 | -6.70 (7.31) | 125 | 4.14 | 0.07 |
| rs2301109 | 62197596 | G | Regulatory | 0.99 | -0.84 (1.75) | | 0.54 | -6.70 (7.31) | 125 | 12.55 | 0.13 |
| rs7143164 | 62166755 | C | Intergenic | 0.99 | 0.97 (1.86) | | 0.54 | -10.06 (7.95) | 104 | 0.32 | 0.21 |
| rs12232182 | 62176220 | C | Transcript | 0.99 | 0.42 (1.08) | | 0.54 | -5.73 (4.58) | 124 | 12.60 | 0.17 |
| rs10148514 | 62187821 | C | Regulatory | 0.99 | 0.57 (2.65) | | 0.54 | -11.70 (11.13) | 124 | 2.90 | 0.18 |
| rs11622129 | 62198642 | T | Intergenic | 0.99 | 0.14 (1.18) | | 0.59 | -3.44 (4.97) | 125 | 0.97 | 0.05 |
| rs11158358 | 62198954 | G | Intergenic | 0.99 | 0.14 (1.18) | | 0.59 | -3.44 (4.97) | 125 | 1.02 | 0.04 |
| rs2301110 | 62199955 | C | Intergenic | 0.99 | 0.14 (1.18) | | 0.59 | -3.44 (4.97) | 125 | 4.85 | 0.07 |
| rs3783752 | 62185692 | A | Transcript | 0.99 | -0.85 (2.38) | | 0.54 | -9.05 (9.86) | 105 | 3.67 | 0.18 |
| rs35244739 | 62211111 | C | Transcript | 0.99 | -0.26 (1.08) | | 0.61 | -2.54 (4.87) | 95 | 1.20 | 0.10 |

*HIF1α*, hypoxia inducible factor-1-alpha; RR - relative risk; RVEF – right ventricular ejection fraction; RVEDVI – indexed right ventricular end-diastolic volume; SE - standard error; SNP, single nucleotide polymorphism; TSS - transcription start site

^a^ Each white and gray block represent variants in linkage disequilibrium (i.e., correlated) based on LDlink's SNPclip (r^2^=0.8, MAF=0.01). The top three SNPs are at least 80% correlated and bottom three SNPs are at least 80% correlated.

^b^ Position is based on the information from Genome Reference Consortium Human Build 37 (GRCh37) (also known as hg19)

^c^ P-values are adjusted using false discovery rate to account for multiple testing

^d^ Adjusted for time between surgical repair and the first CMR, time between the first and second CMR

^e^ Scaled Combined Annotation Dependent Depletion (CADD) - variants with CADD>10 are predicted to fall in the top 10% of the most deleterious variants in the genome

^f^ Genome-Wide Annotation of Variants (GWAVA) score - predicts the functional impact of non-coding variants/regions (range 0-1)

**Supplementary Table 2** Associations between SNPs^a^ within *HIF1α* and fibrotic load among white cases with repaired tetralogy of Fallot

| SNP | Position^b^ | Risk allele | Function | Square root fibrotic volume | | | Fibrotic score | | N | CADD score ^e^ | GWAVA TSS score^f^ |
| --- | --- | --- | --- | --- | --- | --- | --- | --- | --- | --- | --- |
|  |  |  |  | p-value^c^ | | β (SE)^d^ | p-value^c^ | RR (95% CI)^d^ |  |  |  |
| rs2301106 | 62166563 | C | Intergenic | 0.64 | 0.03 (0.06) | | 0.08 | 1.23 (1.02, 1.48) | 125 | 17.57 | 0.39 |
| rs78703581 | 62184128 | A | Transcript | 0.64 | 0.03 (0.06) | | 0.08 | 1.23 (1.02, 1.48) | 125 | 3.84 | 0.24 |
| rs11851112 | 62170340 | T | Transcript | 0.76 | 0.02 (0.06) | | 0.08 | 1.23 (1.02, 1.48) | 124 | 0.02 | 0.20 |
| rs6573399 | 62215725 | T | Intergenic | 0.84 | 0.01 (0.07) | | 0.053 | 1.33 (1.07, 1.64) | 104 | 4.06 | 0.14 |
| rs76308410 | 62171263 | T | Intronic | 0.29 | 0.09 (0.08) | | 0.04 | 1.43 (1.14, 1.79) | 124 | 11.49 | 0.17 |
| rs11549465 | 62207557 | T | Missense | 0.24 | 0.12 (0.08) | | 0.04 | 1.43 (1.14, 1.78) | 125 | 21.20 | 0.31 |
| rs74481028 | 62213060 | G | Intronic | 0.16 | 0.14 (0.08) | | 0.04 | 1.37 (1.11, 1.70) | 125 | 0.12 | 0.13 |
| rs7161527 | 62202799 | T | Intronic | 0.12 | 0.14 (0.07) | | 0.04 | 1.33 (1.09, 1.62) | 125 | 1.58 | 0.11 |
| rs10147275 | 62213553 | T | Intronic | 0.12 | 0.14 (0.07) | | 0.04 | 1.33 (1.09, 1.62) | 125 | 0.01 | 0.15 |
| rs2057482 | 62213848 | T | Regulatory | 0.12 | 0.14 (0.07) | | 0.04 | 1.33 (1.09, 1.62) | 125 | 8.57 | 0.57 |
| rs1951795 | 62171426 | A | Transcript | 0.12 | 0.15 (0.06) | | 0.053 | 1.26 (1.06, 1.51) | 125 | 12.38 | 0.27 |
| rs12435848 | 62176891 | G | Intergenic | 0.16 | 0.09 (0.05) | | 0.08 | 1.19 (1.01, 1.40) | 125 | 2.65 | 0.24 |
| rs12891737 | 62196186 | C | Intergenic | 0.16 | 0.09 (0.05) | | 0.08 | 1.20 (1.01, 1.42) | 119 | 3.09 | 0.03 |
| rs12435848 | 62176891 | A | Transcript | 0.12 | 0.17 (0.06) | | 0.051 | 1.28 (1.07, 1.53) | 124 | 2.71 | 0.24 |
| rs2301111 | 62200201 | G | Intergenic | 0.16 | 0.09 (0.05) | | 0.08 | 1.19 (1.01, 1.40) | 125 | 7.99 | 0.13 |
| rs75606298 | 62201500 | G | Transcript | 0.16 | 0.09 (0.05) | | 0.08 | 1.19 (1.01, 1.40) | 125 | 6.22 | 0.11 |
| rs4899057 | 62202942 | G | Transcript | 0.12 | 0.15 (0.06) | | 0.053 | 1.26 (1.06, 1.51) | 125 | 0.10 | 0.06 |
| rs10873142 | 62203462 | C | Transcript | 0.12 | 0.14 (0.06) | | 0.06 | 1.26 (1.05, 1.52) | 123 | 2.63 | 0.10 |
| rs4902082 | 62212675 | C | Transcript | 0.12 | 0.15 (0.06) | | 0.053 | 1.26 (1.06, 1.51) | 125 | 5.05 | 0.23 |
| rs11621525 | 62203056 | A | Transcript | 0.14 | 0.12 (0.07) | | 0.07 | 1.28 (1.05, 1.58) | 105 | 0.64 | 0.06 |
| rs2301113 | 62206548 | C | Transcript | 0.12 | 0.12 (0.06) | | 0.23 | 1.17 (0.95, 1.43) | 96 | 2.18 | 0.07 |
| rs2301115 | 62206913 | T | Transcript | 0.12 | 0.13 (0.06) | | 0.23 | 1.17 (0.96, 1.44) | 95 | 2.28 | 0.04 |
| rs4902081 | 62208474 | C | Transcript | 0.12 | 0.12 (0.06) | | 0.23 | 1.17 (0.95, 1.43) | 96 | 1.23 | 0.04 |
| rs10135579 | 62173187 | G | Transcript | 0.25 | 0.16 (0.12) | | 0.97 | 1.01 (0.66, 1.54) | 125 | 3.36 | 0.26 |
| rs76276969 | 62173727 | C | Transcript | 0.25 | 0.16 (0.12) | | 0.97 | 1.01 (0.66, 1.54 | 125 | 0.60 | 0.23 |
| rs10129270 | 62181954 | A | Regulatory | 0.16 | 0.18 (0.10) | | 0.97 | 1.01 (0.70, 1.46) | 125 | 3.97 | 0.46 |
| rs77830262 | 62188791 | A | Transcript | 0.25 | 0.16 (0.12) | | 0.97 | 1.01 (0.66, 1.54) | 125 | 2.68 | 0.20 |
| rs10144011 | 62191482 | T | Transcript | 0.25 | 0.16 (0.12) | | 0.97 | 1.01 (0.66, 1.54) | 125 | 13.70 | 0.22 |
|  |  |  |  |  |  | |  |  |  |  |  |
|  |  |  |  |  |  | |  |  |  |  |  |
|  |  |  |  |  |  | |  |  |  |  |  |
| rs12882000 | 62175273 | T | Transcript | 0.12 | 0.16 (0.09) | | 0.84 | 1.06 (0.79, 1.41) | 125 | 11.47 | 0.17 |
| rs12881961 | 62175801 | G | Transcript | 0.12 | 0.16 (0.09) | | 0.84 | 1.06 (0.79, 1.41) | 125 | 0.30 | 0.15 |
| rs10459450 | 62180485 | A | Intergenic | 0.12 | 0.16 (0.09) | | 0.84 | 1.06 (0.79, 1.41) | 125 | 9.58 | 0.20 |
| rs4899056 | 62189531 | T | Transcript | 0.12 | 0.20 (0.08) | | 0.71 | 1.11 (0.85, 1.45) | 125 | 6.65 | 0.14 |
| rs12887165 | 62195945 | C | Intergenic | 0.12 | 0.16 (0.09) | | 0.84 | 1.06 (0.79, 1.41) | 125 | 1.52 | 0.00 |
| rs71423444 | 62196197 | C | Intergenic | 0.17 | 0.14 (0.09) | | 0.87 | 1.05 (0.78, 1.41) | 118 | 1.64 | 0.07 |
| rs67229007 | 62196204 | A | Intergenic | 0.12 | 0.16 (0.09) | | 0.84 | 1.06 (0.79, 1.41) | 125 | 0.47 | 0.07 |
| rs1957757 | 62196948 | T | Intergenic | 0.12 | 0.16 (0.09) | | 0.84 | 1.06 (0.79, 1.41) | 125 | 3.70 | 0.08 |
| rs8005745 | 62185295 | T | Transcript | 0.12 | 0.19 (0.08) | | 0.84 | 1.08 (0.82, 1.43) | 124 | 3.24 | 0.17 |
| rs2284999 | 62186478 | C | Transcript | 0.12 | 0.19 (0.08) | | 0.84 | 1.08 (0.82, 1.43) | 124 | 13.51 | 0.19 |
| rs2301108 | 62197464 | A | Intergenic | 0.12 | 0.16 (0.09) | | 0.84 | 1.06 (0.79, 1.41) | 125 | 4.14 | 0.07 |
| rs2301109 | 62197596 | G | Regulatory | 0.12 | 0.16 (0.09) | | 0.84 | 1.06 (0.79, 1.41) | 125 | 12.55 | 0.13 |
| rs7143164 | 62166755 | C | Intergenic | 0.16 | 0.17 (0.10) | | 0.97 | 0.98 (0.68, 1.39) | 104 | 0.32 | 0.21 |
| rs12232182 | 62176220 | C | Transcript | 0.12 | 0.11 (0.05) | | 0.08 | 1.21 (1.03, 1.43) | 124 | 12.60 | 0.17 |
| rs10148514 | 62187821 | C | Regulatory | 0.12 | 0.29 (0.13) | | 0.69 | 1.18 (0.78, 1.78) | 124 | 2.90 | 0.18 |
| rs11622129 | 62198642 | T | Intergenic | 0.29 | 0.07 (0.06) | | 0.08 | 1.21 (1.02, 1.44) | 125 | 0.97 | 0.05 |
| rs11158358 | 62198954 | G | Intergenic | 0.29 | 0.07 (0.06) | | 0.08 | 1.21 (1.02, 1.44) | 125 | 1.02 | 0.04 |
| rs2301110 | 62199955 | C | Intergenic | 0.29 | 0.07 (0.06) | | 0.08 | 1.21 (1.02, 1.44) | 125 | 4.85 | 0.07 |
| rs3783752 | 62185692 | A | Transcript | 0.24 | 0.16 (0.12) | | 0.87 | 0.93 (0.58, 1.49) | 105 | 3.67 | 0.18 |
| rs35244739 | 62211111 | C | Transcript | 0.25 | 0.08 (0.06) | | 0.27 | 1.14 (0.95, 1.38) | 95 | 1.20 | 0.10 |

*HIF1α*, hypoxia inducible factor-1-alpha; RR, relative risk; SE, standard error; SNP, single nucleotide polymorphism; TSS, transcription start site

^a^ Each white and gray block represent variants in linkage disequilibrium (i.e., correlated) based on LDlink's SNPclip (r^2^=0.8, MAF=0.01). The top three SNPs are at least 80% correlated and bottom three SNPs are at least 80% correlated.

^b^ Position is based on the information from Genome Reference Consortium Human Build 37 (GRCh37) (also known as hg19)

^c^ P-values are adjusted using false discovery rate to account for multiple testing

^d^ Adjusted for time between surgical repair and the first CMR, time between the first and second CMR

^e^ Scaled Combined Annotation Dependent Depletion (CADD) - variants with CADD>10 are predicted to fall in the top 10% of the most deleterious variants in the genome

^f^ Genome-Wide Annotation of Variants (GWAVA) score - predicts the functional impact of non-coding variants/regions (range 0-1)
